# In Vitro Embryogenesis and Gastrulation Using Stem Cells in Mice and Humans

**DOI:** 10.3390/ijms241713655

**Published:** 2023-09-04

**Authors:** Seung Yeon Oh, Seung Bin Na, Yoo Kyung Kang, Jeong Tae Do

**Affiliations:** Department of Stem Cell Regenerative Biotechnology, Konkuk Institute of Technology, Konkuk University, Seoul 05029, Republic of Korea; osy3140@gmail.com (S.Y.O.); amanda1220@naver.com (S.B.N.); aeki0324@naver.com (Y.K.K.)

**Keywords:** synthetic embryo, embryogenesis, gastrulation, stem cells

## Abstract

During early mammalian embryonic development, fertilized one-cell embryos develop into pre-implantation blastocysts and subsequently establish three germ layers through gastrulation during post-implantation development. In recent years, stem cells have emerged as a powerful tool to study embryogenesis and gastrulation without the need for eggs, allowing for the generation of embryo-like structures known as synthetic embryos or embryoids. These in vitro models closely resemble early embryos in terms of morphology and gene expression and provide a faithful recapitulation of early pre- and post-implantation embryonic development. Synthetic embryos can be generated through a combinatorial culture of three blastocyst-derived stem cell types, such as embryonic stem cells, trophoblast stem cells, and extraembryonic endoderm cells, or totipotent-like stem cells alone. This review provides an overview of the progress and various approaches in studying in vitro embryogenesis and gastrulation in mice and humans using stem cells. Furthermore, recent findings and breakthroughs in synthetic embryos and gastruloids are outlined. Despite ethical considerations, synthetic embryo models hold promise for understanding mammalian (including humans) embryonic development and have potential implications for regenerative medicine and developmental research.

## 1. Introduction

Early embryonic development is the initial step toward the formation of the complete organism. Fertilized embryos develop to the pre-implantation blastocyst stage, composed of epiblast (EPI), primitive endoderm (PrE), and trophectoderm (TE) [1]. Following implantation, blastocysts undergo gastrulation, which marks a series of orchestrated cellular events and the onset of the three germ layers—ectoderm, mesoderm, and endoderm—giving rise to numerous tissues and organs. Although pre-implantation development can be recapitulated through in vitro fertilization and cultivation, there are limitations in recapitulating the entire process of embryonic development in vitro, including the complex structures, implantation, and gastrulation [2,3,4,5,6].

In recent years, stem cells have emerged as a powerful tool for studying embryogenesis and gastrulation. Pre- and post-implantation embryo-like structures (synthetic embryos or embryoids), including blastocyst-like structures (blastoids), can be generated in vitro by co-culturing cells derived from each lineage of the blastocysts, including embryonic stem cells (ESCs) and trophoblast stem cells (TSCs), which are in vivo counterparts of EPI and trophectoderm, respectively [7]. The addition of extraembryonic endoderm cells (XENCs), counterparts of primitive endoderm, enables synthetic embryos to expand and mimic embryogenesis with greater accuracy [8]. These in vitro embryo-like structures exhibit greater similarities to peri-implantation embryos in terms of morphological development and gene expression, thus offering a faithful recapitulation of the early embryonic developmental process. Blastocysts and peri-implantation stage-like embryos could be generated by in vitro three-dimensional (3D) culture by culturing aggregates of embryonic and extraembryonic lineage stem cells, such as ESCs, TSCs, and XENCs [7,8]. ESCs and TSCs contribute to EPIs and trophoblast lineages, and the layer of XENCs supports embryo maturation by providing regionalization cues for cell polarization and the maturation of embryonic/extraembryonic compartments [9,10].

Remarkably, even individual cell types can be harnessed to generate synthetic embryos. For instance, ESCs that contain dox-inducible Gata4/6 or Cdx2 could serve as substitutes for the TSCs and XENCs during embryoid formation [11,12,13,14,15]. These models are specifically intended to replicate the later stages of embryonic development that go beyond mid-gastrulation [6,12,15,16]. Totipotent-like stem cells, which are capable of giving rise to both embryonic and extraembryonic lineages upon differentiation, can be used for the generation of synthetic embryos. Blastoids formed from totipotent-like stem cells exhibit similarities to early-stage pre-implantation embryos and could be implanted into the uteruses of surrogate mothers. Furthermore, they could develop into embryo-like structures, forming amniotic cavities through in vitro culture (IVC) [17,18,19,20,21,22,23].

Understanding the complex mechanisms underlying embryogenesis and gastrulation has been a longstanding goal in developmental biology, as it holds great potential for advancing regenerative medicine and understanding developmental and birth defects in humans. The utilization of human stem cells to generate human embryonic models offers an alternative avenue to study human embryonic development. Human ESCs and induced pluripotent stem cells (iPSCs) have been used to study in vitro early embryonic development by forming human blastoids [24,25,26,27,28]. These blastoids effectively mimic they key aspects of pre-implantation development at E5–7, and their further cultivation has demonstrated the potential for developing post-implantation tissues. However, the study of human blastoids beyond E14 is restricted due to stringent regulations on human embryo research [29]. Consequently, it is anticipated that further research focusing on gastruloids capable of partially replicating developmental stages after E14 will pave the way for exploring the later embryonic developmental stages.

In this review, we aim to provide a comprehensive overview of the progress made in studying in vitro embryogenesis and gastrulation using stem cells. We explore the various approaches employed to derive in vitro embryo models and manipulate stem cells to investigate these intricate processes. Furthermore, this review outlines recent findings and breakthroughs in synthetic embryos and summarizes the more practical and specific applications of these techniques.

## 2. Early Embryonic Development in Mice

### 2.1. Pre-Implantation Embryonic Development

**Figure 1:**
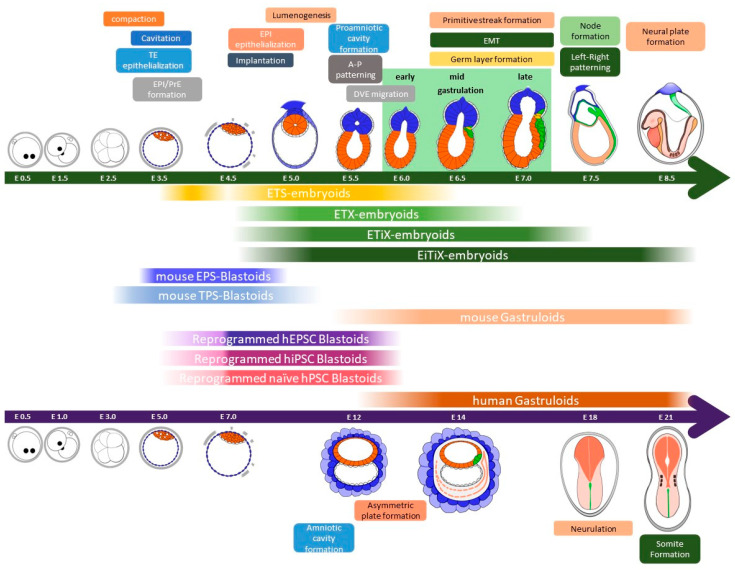
Summary of the early developmental process in natural mouse and human embryos and the corresponding developmental potency of the various synthetic embryos and gastruloids reported. The upper illustration represents the developmental stages of mouse embryos at a specific day of development (E0.5–8.5), while the lower figure illustrates the developmental stages of human embryos at a specific day of development (E0.5–21). The range of developmental potential of each embryoid and gastruloid is indicated by the start and end points of the box. TE: Trophectoderm; EPI: Epiblast; PrE: Primitive endoderm; A-P: Anterior–Posterior; DVE: Distal visceral endoderm; EMT: Epithelial–mesenchymal transition.

In mammals, embryogenesis begins with fertilization, i.e., the fusion of a sperm and egg, resulting in the formation of a zygote as depicted in Figure 1 [1]. Terminally differentiated germ cells, sperm, and oocytes reestablish totipotency through fertilization [30]. Totipotency is maintained until the two-cell stage, and only a small proportion of blastomeres in the four-cell embryos retain totipotency [30,31,32]. The zygote undergoes cell cleavage, resulting in the compacted morula that is composed of 8–16 cells at E2.5 [1]. During compaction, the contact area between blastomeres increases and flattens, which promotes the formation of tight junctions between adjacent outward-facing cells [33,34,35]. Simultaneously, these cell shape changes are regulated by the extension of long filopodia, dependent on E-cadherin, which connects neighboring cells. As the embryo progresses to the 16 to 32-cell stage, the outer cells, trophectoderm epithelium, transport fluid into the intercellular spaces along an ion gradient established by the Na^+^-K^+^ ion pump. This process forms a fluid-filled cavity called the blastocoel [35]. At the end of the morula stage, asymmetrical cell lineage differentiation occurs, resulting in the division of cells into two distinct lineages that make up the blastocyst: the inner cell mass (ICM) and the outer trophectoderm (TE) [8]. Several lineage selector genes, such as *Oct4*, *Nanog*, *Cdx2*, and *Gata6*, are associated with the fate decision.

The outer cells of the morula begin to exhibit a decreased expression of *Oct4* and *Nanog* while initiating the expression of *Cdx2*, which gives rise to TE. In contrast, inner cells expressing *Oct4* and *Nanog* give rise to ICM [36,37,38]. The expression of *Cdx2* is regulated by the Hippo/Yes-associated protein (YAP) pathway [39,40]. In the outer cells, the hippo signaling pathway is attenuated, leading to the translocation of YAP proteins into the nucleus, where they bind to Tead4 [1,40]. Tead4, along with its cofactor YAP, induces the expression of *Cdx2*. Conversely, in the inner cells, the Hippo pathway is activated, leading to the activation of Lats kinase, which allows for the phosphorylation of YAP. Phosphorylated YAP is subsequently degraded through ubiquitination, resulting in the suppression of the expression of *Cdx2* [1,40].

During the subsequent development into the late stage of the blastocyst, the ICM further differentiates into two distinct types: EPI and primitive endoderm (PrE). EPI cells are localized in the inner part of the ICM, and PrE are localized adjacent to the blastocoel cavity. Unlike the ICM and TE specifications, the lineage specification of the EPI and PrE is not determined by the position of the cells within the ICM. Among the cells in the ICM, cells expressing *Gata6* migrate to the vicinity of the blastocoel cavity and form PrE. Meanwhile, cells that continuously express *Oct4* and *Nanog* migrate inward and become EPI cells [37]. Finally, E4.5 pre-implantation blastocysts consist of three cell lineages: EPI, TE, and PrE [1].

### 2.2. Peri- and Post-Implantation Embryonic Development

At E4.5, the EPI cells of the peri-implantation blastocyst undergo a transformation that adopts a wedge-shaped morphology and forms a rosette-like structure [41]. In pre-implantation embryos, the cell membrane exhibits a ubiquitous expression of E-cadherin, but in post-implantation embryos, E-cadherin becomes localized to the apical region of the rosette-like EPI [41]. This apical region gives rise to a proamniotic cavity and is accompanied by the expression of anti-adhesive molecules such as Podocalyxin. The epithelialization of the EPI occurs simultaneously with the formation of the proamniotic cavity [41]. At E5.0–6.0, the mouse embryo undergoes rapid enlargement, and the elongated cavity, known as the egg cylinder, provides a platform for the proliferation of the EPI.

### 2.3. Gastrulation

At E6.25–6.5, a primitive streak is formed at the posterior portion of the EPI originating from a node, and a structure guides the development of the primitive streak and produces molecular signals such as Nodal and fibroblast growth factor (FGF). Nodal, a member of the TGF-β superfamily, starts to be expressed in the ICM of the E3.5 blastocyst [42,43]. Nodal signaling plays a crucial role in the nascent EPI by orchestrating the precise patterning of the visceral endoderm (VE), formation of the anterior–posterior (A-P) axis, and development of the extraembryonic ectoderm (ExE) [44]. Nodal is essential for the proper development of the anterior head structure in the hindbrain, as demonstrated by chimeric embryos with knocked-out primitive endoderm [42]. In the posterior region, Wnt and Nodal signals are induced by Bmp4 signals originating from the ExE [7]. Around E6.25, as the primitive streak begins to develop at the proximal posterior EPI, cells undergo an epithelial-to-mesenchymal transition (EMT). EMT is regulated by the Wnt, Bmp, FGF, and Nodal signaling pathways [45,46,47,48,49,50].

Starting from primitive streak formation, gastrulation continues until the formation of three primary germ layers: ectoderm, mesoderm, and endoderm. EPI cells move down to the underlying layers and eventually form the mesoderm and definitive endoderm at around E7.0. As they migrate to the anterior tip of the primitive streak, the EPI cells give rise to the axial mesendoderm, which includes the definitive endoderm, notochord, and node [36]. The node, which is a transient structure appearing at E7.5 and disappearing at E9.0, contributes to the formation of the notochord [51,52]. Once the notochord is placed along the embryonic axis, it serves as a structural center as well as a regulator in patterning the neural tube and establishing the dorsoventral (DV) axis within the central nervous system (CNS) [53,54]. The EPI that does not migrate through the primitive streak undergoes differentiation into the neuroectoderm (NE), representing the default state of EPI differentiation [55,56,57]. After gastrulation, the newly allocated cells differentiate into precursors of various organs and tissues based on their respective germ layers.

In addition to somatic cells, germ cells are formed from EPI. Primordial germ cell (PGC) precursors are found in the proximal EPI around E6.0, before the onset of gastrulation [58]. PGC precursors are formed by reacting with Bmp4 and Bmp8b signals secreted from the ExE. Therefore, cells expressing BMP receptors and Smad, which is a downstream signaling molecule of BMP, become PGC precursors. The Wnt signaling pathway also regulates PGC signaling [59,60,61,62,63,64]. These PGC precursors migrate to the posterior EPI region and form clusters at E7.25. By forming these clusters, the precursors become PGCs. Previous gene knockout studies have shown that bone morphogenetic protein (Bmp) signals are essential for PGC generation in EPI.

## 3. Synthetic Embryos Constructed with Mouse ESCs and TSCs (ETS-Embryoids)

There have been many attempts to mimic early embryonic development to understand the mechanism of embryogenesis and implantation in in vitro systems. In vitro studies of pre-implantation development can be conducted using fertilized embryos. However, these studies also can be conducted without oocytes and sperm, although there are limitations in recapitulating the entire process of embryonic development in vitro [2,3,4,5,6]. Recently, stem cell technology has achieved the successful generation of blastocyst- and embryo-like structures in an egg-free system. These embryo-like structures, generated by the combination of stem cells without eggs, are referred to as synthetic embryos.

**Figure 2:**
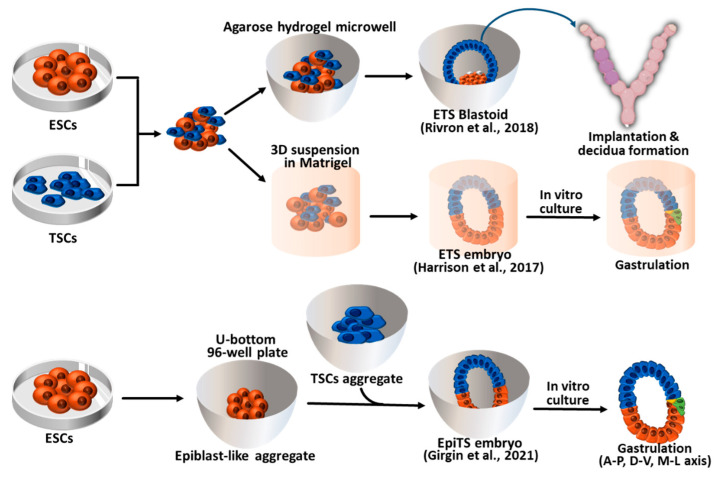
Synthetic embryos constructed with mouse ESCs and TSCs. ETS blastoids/embryos and EpiTS embryos were generated from the aggregates of ESCs and TSCs. ETS blastoids can be implanted into the surrogate mother and form decidua. Both ETS- and EpiTS-embryos can progress to the gastrula stage embryos through an in vitro culture system. ESCs: Embryonic stem cells; TSCs: Trophoblast stem cell; A-P: Anterior–Posterior; D-V: Dorsal–Ventral; M-L: Medio–Lateral [7,10,72].

Pre-implantation blastocysts consist of three cell layers, namely EPI, TE, and PrE. Each of these cell types can be established as stem cell types in vitro. EPI cells give rise to pluripotent embryonic stem cells (ESCs) in vitro under LIF-Stat3 signaling activation. The other two types of extraembryonic cells, TE and PrE, can also be established as trophoblast stem cells (TSCs) and extraembryonic endoderm cells (XENCs), respectively [65,66,67]. Researchers have attempted to recapitulate pre- and post-implantation embryos using three different types of blastocyst-derived stem cells, specifically ESCs, TSCs, and XENCs. In 2017, an initial trial was conducted by Zernicka-Goetz et al. using only two types of stem cells: ESCs and TSCs. These two stem cell types were combined to generate synthetic embryos which they referred to as “ETS embryos” in Figure 2 [7]. The researchers placed single ESCs and small clumps of TSCs in Matrigel (as a substitute for the PrE) and cultured them in a medium that allowed for the development of ESCs and TSCs. After 96 h of culture, the ETS embryos reached a size of 100 μm × 200 μm, and the number of cells and their morphology were similar to those of natural E5.5 embryos. In the development of ETS embryos, the formation of the proamniotic cavity faithfully recapitulated that of natural embryos. The ESC and TSC cavities formed separately, and at 96 h of ETS embryo development, these cavities merged into a single cavity. Using this in vitro embryogenesis system, Zernicka-Goetz et al. found that the Activin/TGF-β signal originating from the ESC compartment played a crucial role in the formation of TSC cavitation in both ETS and natural embryos. These ETS embryos further developed into E6.5 embryo-like structures showing mesoderm formation and primordial germ cell (PGC) specification. The ETS-embryos, despite not having enough PrE cells to develop into the distal visceral endoderm (DVE) and anterior VE (AVE), still underwent symmetry breaking for gastrulation [7]. After 100 h of culture, neighboring TSC compartments promoted the induction of *Brachyury*, a mesodermal marker [68], in the ESC compartment as E6.5 natural embryo [69]. When cultured for 120 h, cells expressing Tfap2c, Oct4, and stella were detected in the area overlapping T/Brachyury-expressed location, indicating PGC specification [70,71].

In 2018, Rivron et al. generated a blastocyst-like structure termed ETS-blastoid by using ESCs and TSCs. To facilitate the development of blastoids, the researchers employed agarose hydrogel microwells to facilitate the formation of non-adherent aggregates [72,73]. Through the aggregation of five ESCs and subsequently adding twelve TSCs after 24 h of aggregation, these aggregates efficiently developed into blastocyst-like structures measuring 90 μm in diameter following a culture period of 65 h [72]. The ETS-blastoids enabled PrE-like cell formation and were morphologically and transcriptionally similar to E3.5 blastocysts, even in the absence of XENCs or extracellular matrix scaffolds. Notably, the implantation of a mouse embryo can be reproduced using ETS-blastoids after transferring them into the pseudo-pregnant female uterus. Although these ETS-blastoids failed to develop embryonic lineage, they showed few signs of extraembryonic development (giant cell formation and the expression of post-implantation trophoblast markers) and decidualization (vascular anastomosis and *ALDH3A1* expression in the decidua). Using this in vitro implantation system, they found interacting signaling pathways that are important for implantation. FGF4 (from embryos) [65] and IL-11 (from both embryonic and trophectoderm compartments) [74] regulate trophoblast proliferation/self-renewal and *Cdx2* expression. BMP4 and Nodal originating from the embryonic compartment [43,45] and WNT6 and WNT7B originating from trophectoderm [75] contribute to trophectoderm maturation and morphogenesis, supporting in utero implantation.

In 2021, an improved version of the ETS model called EpiTS was introduced to enable the realization of gastrulation initiation and axial morphogenesis using a high-throughput approach [10]. In this model, ESC aggregates forming EPI-like aggregates and TSC aggregates were generated separately and merged together. EPI-like and TSC aggregates were cultured for 72–75 h before being merged in a U-bottomed 96-well plate (low-attachment). After 96–120 h of culture, the merged EPI-like/TSC aggregates formed gastrulating embryo-like structures called EpiTS embryoids and underwent mesodermal differentiation and PGC progenitor specification. Notably, they found that EPI epithelialization, along with co-culture with TSC aggregates, is essential for the spatiotemporal recapitulation of the mesodermal differentiation (measured by Brachyury expression) of EPI, indicating the importance of physical interactions between EPI and TSC aggregates. When cultured for 168 h, EpiTS embryoids spontaneously underwent axial morphogenesis, including the anterior–posterior (localization of neurons away from the WNT-positive tip), dorsal–ventral (polarized expression of ectoderm and mesoderm tissues), and medio–lateral (neural tube flanked by paraxial mesoderm) axes.

Collectively, these ETS models can serve as a valuable tool for studying embryo cavitation, axis morphogenesis, and implantation through the co-culture of ESCs and TSCs. However, this ETS model has limitations when it comes to further gastrulation, and the absence of PrE lineage cells restricts the replication of very early embryogenesis. Consequently, there is a growing demand for a new model that can address these challenges and provide a more comprehensive understanding of embryonic development.

## 4. Synthetic Embryos Constructed with ESCs, TSCs, and XENCs

**Figure 3:**
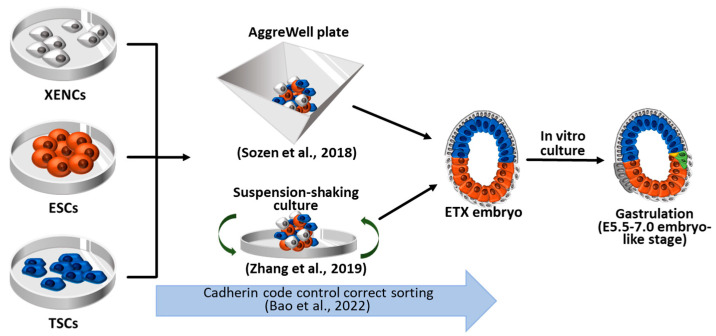
Synthetic embryos constructed with wild-type mouse ESCs, TSCs, and XENCs (called ETX embryos or ETX-embryoids). The self-organization and sorting of ESCs, TSCs, and XENCs are controlled by cadherin code, facilitating the formation of ETX-embryoids. These ETX-embryoids can develop to the stage comparable to natural embryos at E5.5–7.0. ESCs: Embryonic stem cells; TSCs: Trophoblast stem cells; XENCs: Extraembryonic endoderm cells [6,8,78].

Since ETS methods were relatively inefficient and the resulting embryos yielded an insufficient amount of PrE lineages necessary for VE formation, Sozen et al. introduced an advanced approach, named ETX embryos, where they incorporated XENCs into the ETS model as illustrated in Figure 3 [8]. XENCs are derived from the PrE of blastocysts, expressing unique markers for extra-embryonic endoderm derivates, and they can contribute exclusively to extra-embryonic endoderm lineages [76]. By E5.0, the PrE undergoes segregation into two subpopulations—VE and parietal endoderm (PE)—that play crucial roles in embryonic development, patterning, and maturation [77]. The inclusion of XENCs not only results in a closer resemblance to the structure of a natural embryo but also enables the formation of an embryo-like structure without the need for an external extracellular matrix (ECM) supply, such as Matrigel. Moreover, this approach allows for the investigation of further developmental stages that were not able to be studied using the ETS model. To enhance efficiency and promote post-implantation development, several approaches have been suggested based on the co-culturing model using ETX embryos [6,9,10,11,12,13,15]. These ETX-embryoids recapitulated the developmental events observed in E4.5–E7.0 natural embryos, including lumenogenesis for the formation of a proamniotic cavity (PCX, E-CAD, and aPKC expression) [6], asymmetry breaking (T/Brachyury expression), and PGC specification, as shown in ETS-embryos. However, the ETX-embryoids demonstrate more similar cell proportions to natural embryos and undergo further post-implantation development [8].

### 4.1. ETX Embryos Using Wild-Type ESCs, TSCs, and XENCs

The primary distinction between the ETS-model and the ETX model lies in the presence of a layer of XENC-like cells that generate a VE-like structure that induces signals for lumenogenesis, A-P axis, and EMT [79,80]. On day 5 of culture with XENCs, the ESC compartment overlaying XENCs becomes squamous, and the TSC compartment overlaying XENCs becomes cuboidal [8], resembling the E6.75 natural embryos [81]. Supplementing the XENC layer is essential for the maturation of both the ESC and TSS compartments by providing a basal membrane, and it plays a crucial role in embryonic development [82]. However, it should be noted that XENCs are unable to contribute to PE, resulting in the inevitable absence of parietal yolk sac formation [77].

ETS-embryos need ECM as a substitute for PrE lineages. However, ECM and adherent environments are not required for the generation of ETX-embryos [6,8,12,13,15,16]. Therefore, shaking, rolling, or rotating culture systems can be adapted for ETX-embryo formation with higher efficiency or enhanced development [6,12,15,16]. After 4 days, aggregates of ESCs, TSCs, and XENCs within inverted-pyramidal microwell (AggreWell) plates generate a structure resembling E5–6 mouse embryos, wherein the ESC and the TSC compartments merge with each other and are enveloped by a cell layer derived from XENC [8]. Zhang et al. compared several different methods for ETX embryo formation and established the suspension-shaking culture method, which was most suitable for stimulating intercellular communication, recognition, and organization into specific compartments in order [6].

Sozen et al. used AggreWell plates to form aggregates of the three types of blastocyst-derived stem cells, namely ESCs, TSCs, and XENCs, thereby generating ETX-embryos [8] The ETX system enabled the researchers to recapitulate the in vitro processes of AVE formation, EMT, mesoderm, PGCs, and definitive endoderm formation, as observed during early post-implantation development. After 96 h of culture, the ETX-embryos developed into a structure resembling E5.5 natural embryos. When comparing the developmental potential of ETX- and ETS-embryos, ETX-embryos yielded more T/Brachyury-expressing cells than ETS-embryos. These T/Brachyury-expressing cells were found to be expressing EMT markers, *Snail*, *Vimentin*, *Mmp9*, and *N-cadherins* [83]. Intriguingly, PGC-like cells emerged on day 6 of culture at the boundary between the ESC and TSC compartments, analogous to the location where mesoderm and PGC are formed in E6.5–7.0 natural embryos. AVE specification in ETX-embryos was confirmed by the asymmetric expression of Lefty1 on days 4–5 of culture and the expression of *Otx2* (a regulator of AVE), which was confined to XENCs overlying the EPI region but not in the extraembryonic compartment. Definitive endoderm cells derived from ESCs, characterized by *Foxa2* and *Sox17* expression, emerged on day 5 and eventually replaced the VE layers derived from XENCs by the end of day 6. Collectively, the ETX-embryos generated by Sozen et al. faithfully recapitulated the spatio-temporal events observed during gastrulation in natural embryos.

Zhang et al. utilized a nonadherent-suspension-shaking method to culture a combination of three types of stem cells—ESCs, TSCs, and XENCs—and generated embryo-like structures resembling E5.75 embryos [6]. These ETX-embryoids exhibited the spatial distribution of extraembryonic ectoderm, EPI, and VE and formed a proamniotic cavity. Cavities formed earlier in the ESC compartment (by 60 h) than in the TSC compartment (by 72 h) and later coalesced to form a large cavity (by 84 h). To trace the XENC contribution in the ETX-embryoids, they used Lefty1-mCherry transgenic XENCs and found that DVE/AVE markers were detected in mCherry^+^ XENC-derived cells. By using Blimp1-mVenus and Stella-ECFP double-transgenic ESCs [84,85], PGC specification and mesodermal differentiation could be monitored because PGC marker Blimp1 is also co-expressed in many mesoderm-involved genes [86,87]. At 84 h, Blimp1-mVenus^+^/Stella-ECFP^−^ cells were detected in the ESC compartment near the ESC and TSC boundary. These Blimp1-mVenus^+^/Stella-ECFP^−^ cells were found to express other PGC markers (*Prdm14*, *Stella*, and *Tfapc2*) and mesoderm markers (*Flk1*, *Hhex*, and *Hand1*), indicating that mesoderm formation and PGC specification were induced in confined areas of ETX-embryoids. Moreover, the ETX-embryoids showed implantation potential after transfer into E2.5–3.5 pseudopregnant mice. At 48 h post-transfer, E5.5 embryo-like structures were observed within the decidual tissue of the uterus. However, the subsequent examination failed to detect further embryonic morphologies, indicating a limited developmental potential restricted to the earlier stages of implantation.

Bao et al. discovered that the efficiency of ETX-embryoid formation was closely associated with cadherin codes [78]. Three major kinds of cadherins regulate cell–cell adhesion force during embryogenesis and gastrulation [88]. E-cadherin is expressed across all lineages during peri-implantation development, whereas P-cadherin is specifically upregulated in the TE following implantation, and K-cadherin shows elevated levels exclusively in the PrE prior to implantation. Bao et al. observed that only 15.4% of the ETX-embryoids formed correctly sorted structures resembling post-implantation embryos, while a notable proportion exhibited missorted ETX structures such as the mislocalization of XENCs and more than one EPI or trophoblast compartment. They found that the optimal balance between E-cadherin and K-cadherin played a crucial role in ensuring appropriate XENC sorting, and the knock-down of P-cadherin in TSCs or E-cadherin in ESCs disrupted the process of ETX embryogenesis. Overall, the cadherin code exerted a significant influence on sorting efficiency and contributed to enhanced lumenogenesis and basement membrane formation in ETX embryos.

### 4.2. ETiX Embryos Using ESCs Facilitating PrE-Lineage Differentiation

**Figure 4:**
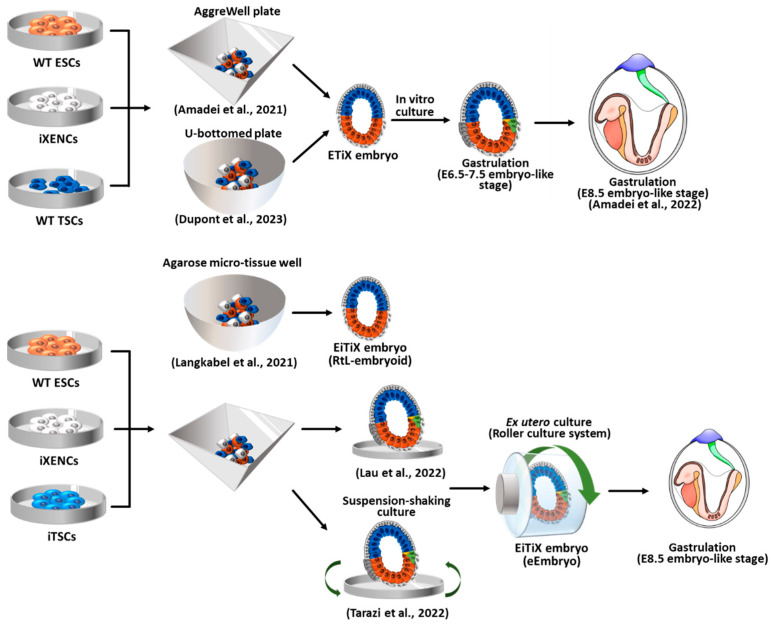
Mouse embryoid formation achieved via combining wild-type ESCs with induced XENCs (iXENCs) and induced TSCs (iTSCs). ETiX embryoids are generated using iXENCs, and EiTiX embryoids are generated using iXENCs and iTSCs. The key events and unique culture methods are illustrated for each EtiX and EiTiX embryoid. WT: wild-type; ESCs: embryonic stem cells; iXENCs: induced extraembryonic endoderm cells; TSCs: trophoblast stem cells; iTSCs: induced trophoblast stem cells [9,11,12,13,15,16].

Since the ETX model system still showed limited gastrulation, several researchers attempted to use cell types other than wild-type stem cells. VE is the PrE-derived cell type that directly interacts with EPI and extraembryonic ectoderm. However, XENCs showed a preferential interaction closer to PE than to VE [89,90]. Therefore, XENCs need to be replaced with more VE-like cell types. The overexpression of *Gata4* or *Gata6* in ESCs efficiently induces endodermal lineage differentiation [91,92], which replaces XENCs (Figure 4) [11,12,13]. Amadei et al. used dox-inducible Gata4-containing ESCs (Gata4-ESCs) as a substitute for XENCs [11,12]. Compacted aggregates were formed 48 h after combining ESCs, TSCs, and dox-treated Gata4-ESCs in AggreWell. Lumenogenesis was observed in ESC and TSC compartments at 72 h, and the lumens were fused at 96 h. These ETX-embryos with induced XENCs were initially termed iETX but are referred to here as ETiX. Four-day-old ETiX embryos resembled E5.5 natural embryos showing AVE specification and migration to the distal/lateral position, which was rarely observed in ETX embryos. On day 5, Amadei et al.’s ETiX embryos showed A-P axis, EMT, mesoderm, and definitive endoderm formation, which typically occurs in E6.5 natural embryos [93,94,95]. In approximately 20% of the ETiX embryos, *Runx1*, an extra-embryonic mesoderm marker, was expressed in T/Brachyury^+^ cells positioned between the VE-like layer and TSC compartment [11,96,97]. However, beyond day 6 of culture, the further development of ETiXs was not possible due to the limitations of the culture environment [11].

Amadei et al. also showed that the ETiX embryos further developed to form neural tubes flanked by somites, a beating heart, gut tubes, yolk sacs, and blood islands, comparable to E8.5 natural embryos [12]. At day 7, T/Brachyury^+^ notochord-like structures were detected in ETiX embryos [12,98]. Notochord induces neurulation by secreting signaling molecules such as the sonic hedgehog molecule to pattern the dorsoventral axis and stimulates neural differentiation [99,100,101]. An SOX1^+^ neural tube-like structure was also found above the notochord, simulating the A-P axis of E8.0 embryos with SOX1^+^ and SOX2^+^ neuroepithelial cells [12,102]. The ETiX embryoids also showed regional expression of PAX6, OLIG2, NKX2-2, FOXA2, PAX3, and SOX10, confirming the presence of the neural tube, floor plate, somatic mesoderm, and neural crest cells [12,103,104,105,106]. Further developmental potential of ETiX embryos was suggested by the detection of subclusters that showed transcriptomic similarity to E9.5 natural embryos [12]. Somite formation, which is crucial for segmental organization, was also observed in ETiX embryoids with paired somite blocks expressing the HOXB4 protein, similar to natural E8.0 embryos [12,107]. Additionally, a beating structure expressing cardiac markers, myosin heavy chain 2 (MYH2), and GATA4 emerged below the encephalon region, reminiscent of the early cardiac development seen in E8.0 natural embryos [108,109]. Amadei et al. attempted to extend the developmental potential of ETiX embryos up to the E8.5 natural embryos by employing the ex utero culture technique on a roller culture platform, as detailed in the study by Aguilera-Castrejon et al. [14]. Although the timing of these developmental events in the ETiX embryoids closely matched the corresponding stages observed in natural embryos, heart looping, which is typically observed in E8.5 natural embryo, was not observed [110]. Day 8 ETiX embryoids reproduced the formation of foregut and hindgut pockets similar to E8.5 natural embryos via expressing SOX2 and SOX17, respectively. However, single-cell RNA-sequencing (scRNA-seq) data revealed that organ-specific identities in the gut appearing after E8.5 were not observed in ETiX, indicating the limited development of the VE [12,95].

ETiX embryoids are also capable of recapitulating the development of extraembryonic structures, including the amnion, yolk sac, and chorion–allantois complex and RUNX1-positive blood islands, mirroring the developmental timeline of natural embryos [12,95,111]. These structures emerged at specific stages; the amnion and amniotic mesoderm appeared on day 6, followed by the yolk sac and allantois on days 7 and 8. However, the chorion lineage in ETiX embryoids exhibited incomplete maturation, as evidenced by an altered or absent expression of genes associated with the ectoplacental cone, trophoblast giant cells, and spongiotrophoblast cells. Therefore, the extraembryonic lineages derived from the EPC were mostly absent in ETiX embryoids, indicating the incomplete replication of extraembryonic development [12]. It is worth noting that the lack of interaction with the maternal environment in the ETiX model may lead to the defective development of the extraembryonic compartment.

To induce the PrE lineage for EtiX embryo formation, Dupont et al. used Gata6-overexpressing ESCs (PrE-ESCs) that contained a dox-inducible *Fgfr2* and *Gata6* transgene [13]. Upon the induction of Gata6 and the stimulation of the FGF-ERK pathway, PrE-ESCs subsequently express *Gata4* and *Sox17*, similar to what is observed during normal embryogenesis [13,112]. Dupont et al. utilized a static culture system, a U-bottomed 384-well plate, and introduced a time-delay method by adding TSCs on the following day after the aggregation of ESCs and PrE-ESCs [13]. This approach led to the ETX-embryos having enhanced development potential, extending it until the late gastrulation stage. Until day 5, the developmental events of Dupont’s EtiX-embryos were comparable to previous ETX or EtiX models, including VE formation, PGC-specification, and mesoderm and A-P axis formation, as observed in E6.0–6.5 natural embryos [13,113]. By day 6, the EtiX-embryos exhibited a structure similar to that of the late gastrulation stage of E7.5 natural embryos, characterized by the formation of exocoelom surrounded by chorion and amnion derived from extraembryonic mesoderm. Staining with Eomes confirmed the presence of a bilayer amnion-like membrane [13,114]. Using whole-mount staining analysis, Tal1^+^ hematoendothelial/blood progenitors-like cells were identified, and this finding was further validated using scRNA-seq, which revealed the similarity between day 6 EtiX and in vivo E7.5 embryos [13,115,116]. The further formation of the headfold, heart, and foregut entrances was sporadically examined in EtiX embryos on day 7 or 8 of culture.

### 4.3. EiTiX Embryos Constructed with ESCs and Induced TSCs (iTSCs) and Induced XENCs (iXENCs)

Langkabel et al. proposed the EiTiX model using ESC and inducible ESC lines (Figure 4) [9,10]. They employed 5F-ESCs (carrying dox-inducible Cdx2, Tfap2c, Eomes, Gata3, and Ets2) as substitutes for TSCs and iGATA6-ESCs (carrying dox-inducible Gata6) as substitutes for XENCs. To achieve a non-adherent 3D culture, these three ESC lines were co-cultured in an agarose micro-tissue well, and after 24 h of culture, doxycycline was added to the medium for 3 days to induce the transgene expression of 5F-ESCs and iGATA6-ESCs. By allowing an additional day without doxycycline, the aggregates successfully underwent compartmentation into ExE-, VE-, and EPI-like structures in a corresponding manner, mimicking E5.25 embryos [3,117]. The formation of rosette and lumen was observed in both the EPI and ExE compartments; however, the fusion of the lumens (leading to the formation of the proamniotic cavity) and further developmental progression were rarely observed. These embryoids were named Rosette-to-Lumen stage embryoids (RtL-embryoids) to highlight their specific transcriptional process, which involves epithelialization to lumenogenesis.

The EiTiX strategy was also employed by the Zernicka-Goetz group in 2022 using iCdx2-ESCs (carrying dox-inducible Cdx2) and iGata4-ESCs (carrying dox-inducible Gata4) (Figure 4) [15]. Aggregates of ESCs, iCdx2-, and iGata4-ESCs were able to form embryo-like structures (called EiTiX-embryoids) resembling post-implantation embryos [15,118,119]. On day 6, EiTiX-embryoids underwent EMT, PGC specification, and the intercalation of definitive endoderm (Foxa2^+^ and Sox17^+^) into the VE-like layer, which corresponded to E6.5–7.5 natural embryos. By day 7 of culture, the EiTiX-embryoids exhibited the A-P axis through the expression of SOX1 and SOX2, indicating the onset of neurulation [102,120]. In addition, the expression of Myh2 and Gata4, accompanied by a beating heart, indicated cardiac development [108,109]. Subsequently, on day 8, the EiTiX-embryoids displayed well-formed headfolds and a developed heart, tail, and chorion, resembling the developmental stage of E8.0–8.5 natural embryos [121,122]. Furthermore, the expression of the pharyngeal mesoderm marker Islet1, which had not been confirmed in previous models, was observed between the heart region and the forebrain region [123]. By tracking the GFP^+^ iCdx2-ESCs, they found that the iCdx2-ESCs had developed into the chorion but not into the ectoplacental cone and trophoblast giant cell lineages.

Tarazi et al. tried to combine past EiTiX-embryoid formation methods (static culture using AggreWells and nonadherent-suspension-shaking method) and an ex utero culture method using a roller culture system to enhance the post-implantation development of EiTiX-embryoids [14,16]. Three naïve pluripotent-state ESCs (wild-type ESCs, iCdx2-ESCs, and iGata4-ESCs) were used to generate EiTiX-embryoids, which subsequently formed egg cylinder-like structures (referred to as “eEmbryos”) after day 5. Upon transfer into a roller ex utero culture system, eight-day-old eEmbryos displayed remarkable similarities to E8.5 natural embryos, including the presence of four pairs of somites, a neural tube, an invaginating foregut, a beating heart, and the establishment of the head-to-tail and dorsoventral axes. Additionally, advanced eEmbryos exhibited blood circulation in the yolk sac. However, like previous embryoid models, the eEmbryos lacked the key markers associated with chorion and ectoplacental cone progenitor cell lineages, such as trophoblast giant cells and spongiotrophoblast cells, despite the emergence of similar structures [14,16].

## 5. Blastoid Formation Using Totipotent-Like Stem Cells

**Figure 5:**
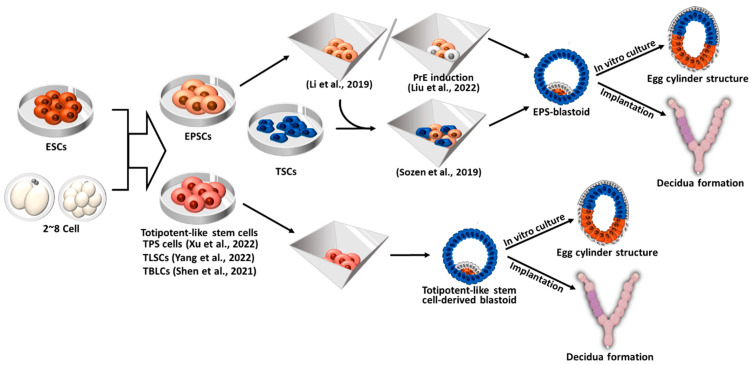
Blastoid formation from mouse expanded potential stem cells (EPSCs) and totipotent-like stem cells. The resulting blastoids displayed the developmental potential to form egg cylinder-like structures through an in vitro culture system and the capacity to induce implantation and decidualization in vivo. EPSCs: expanded potential stem cells; ESCs: embryonic stem cells; TSCs: trophoblast stem cells; TPS cells: totipotent potential stem cells; TLSCs: totipotent-like stem cells; TBLCs: totipotent blastomere-like cells [18,19,20,128,129,130].

ESCs are pluripotent stem cells that can differentiate into all body tissues, except for extraembryonic tissues, under normal conditions. Therefore, ESCs can only form blastoids or embryoids when co-cultured with TSCs and XENCs (ETX-embryoids) or their derivatives (EtiX- and EiTiX-embryoids) [6,7,8,12,13,15]. In contrast, totipotent cells have the ability to differentiate not only into the embryonic but also into extraembryonic tissues. However, true totipotent stem cells do not exist, as the totipotency is observed only in the one-cell embryos and blastomeres at the two-cell stage [124]. Recently, totipotent-like stem cells resembling two-cell embryos have been derived [18,19,20]. Given that a single totipotent cell can self-renew and give rise to EPI, TE, and PrE, totipotent-like stem cells can independently generate blastocyst-like structures (blastoids) without the need for TSCs and XENCs as represented in Figure 5 [125,126,127].

### 5.1. Blastoid Formation from Expanded Potential Stem Cells (EPSCs) and EPS- and EPST-Blastoids

Expanded Potential Stem Cells (EPSCs) are totipotent-like stem cells derived from a single blastomere of eight-cell stage embryos, and they possess the developmental potential to form not only embryonic but also extraembryonic lineages [131]. EPSCs can form dome-like colonies resembling ESCs and exhibit enhanced differentiation potential compared to pluripotent stem cells, as they can differentiate into all somatic cell lineages as well as the trophoblast lineage. EPSCs also can be converted from ESCs or iPSCs by culturing them in EPSC culture medium. Furthermore, EPSCs can be converted into TSCs or XENCs by modifying the culture conditions. Therefore, EPSCs have the ability to generate all types of stem cells originating from blastocysts, enabling them to generate blastoids without the need for TSCs and XENCs [128]. Since EPSCs are in a pluripotent state at the transcriptome level and express high levels of pluripotency markers [132], here, EPSCs are described separately from other totipotent-like stem cells.

Li et al. seeded EPSCs into AggreWell plates, cultured them in KSOM:ETS medium (a combination of KSOM, N2B27, and TSC medium), and supplemented them with FGF4, Heparin, Bmp4 (a TGF-β activator), CHIR99021 (a Wnt activator), and A83-01 (an ALK5 inhibitor), to form blastoids (referred to as EPS-blastoids) [128]. After 5–6 days of cell seeding, the EPS-blastoids exhibited growth and proliferation, resulting in a size and cell number comparable to E3.5 blastocysts (approximately 15% efficiency). EPS-blastoids displayed compaction and polarization events seen in 8- to 16-cell stage embryos; E-cadherin and ZO1 (a tight junction protein) accumulated between the cells, and PAR6 accumulated on the apical surface of the aggregates after 18–72 h of culture [133]. EPS-blastoids developed into egg cylinder-like structures after further culture under IVC conditions and were able to initiate implantation and form decidua after being transferred into pseudopregnant mice [128]. Although retarded and malformed, embryonic (Oct4^+^) and extraembryonic (Eomes^+^ and GATA4^+^) tissues were detected in the EPS-blastoid-derived deciduae.

Recently, Liu et al. found that inducing EPSCs into PrE prior to generating EPS-blastoids increased the efficiency of cavity and blastoid formation [129]. This finding was consistent with a previous report indicating that PrE induction from ESCs prior to blastoid formation supports the survival of EPI and PrE-like cells and improves the efficiency of ETS-blastoid formation [134]. They cultured EPSCs for 2 days in PrE induction medium containing FGF4, retinoic acid, CHIR99021, 8Br-cAMP and before aggregation, resulting in a three-fold increase in the formation efficiency of EPS-blastoids [129]. Liu et al. also suggested that EPS-blastoids showed defective development in TE lineages [129], which contradicts previous studies indicating that EPSCs have an enhanced ability to generate TSC-like cells compared to ESCs [131]. Supporting this discrepancy, Posfai et al. proposed that the in vitro conversion of EPSCs to TSCs was less efficient compared to ESCs [132]. When these EPS-blastoids were further cultured in vitro, they developed into E5.5-like post-implantation embryos containing EPI, VE, ectoplacental cone, and ExE. The EPS-blastoids could be implanted into the pseudopregnant mice and form decidua without normal embryonic tissue [129].

Sozen et al. (2019) generated blastoids by co-culturing EPSCs and TSCs in AggreWell plates under hypoxic conditions (5% O_2_). These blastoids are hereafter referred to as EPST-blastoids [130]. After 4 days of culture, the EPST-blastoids formed structures comparable to mid to late blastocyst stages (E4.5–4.75). After 3 days of IVC beyond implantation, the EPST-blastoids further developed into egg cylinder-like structures surrounded by a PrE-lineage cell layer. Upon implantation into a foster mother, the EPST-blastoids formed deciduae that generated primary and secondary decidual zones equivalent to E7.5 natural decidua. However, the EPST-blastoids failed to form Reichert’s membrane, which protects the fetus in the uterus [135].

### 5.2. Blastoid Formation from Totipotent-Like Stem Cells Other than EPSCs

Totipotent-like stem cells can be derived from two- to eight-cell stage embryos or from pluripotent stem cells through a “pluripotency-to-totipotency transition” [136]. Several research groups have reported different kinds of totipotent-like stem cells which were derived using a different culture medium and different conditions [18,19,20]. These cells are characterized by the expression of two cell-specific genes, namely *Zscan4* and *MERVL*, which contribute to both embryonic and extraembryonic tissue formation and the ability to form blastoids in vitro [127].

Although EPSCs can mimic the process of early pre-implantation development, they are still transcriptionally distinct from two-cell embryos and have less potential for differentiation into the extraembryonic lineages compared to totipotent embryos [132]. To overcome these limitations, Xu et al. explored chemical cocktails to derive totipotent-like stem cells from two-cell stage embryos or convert EPSCs into cells with totipotent potential (referred to as TPS cells) [18]. They found that the inhibition of HDAC1/2 and DOT1L and the activation of RARγ signaling were important for the induction and maintenance of totipotency and established culture medium (CPEC medium) supplemented with CD1530 (RARγ receptor agonist), VPA (HDAC inhibitor), EPZ004777 (DOT1L inhibitor), and CHIR99021 (Wnt signaling agonist). Established TPS cells displayed transcriptomic features similar to those observed in two- to four-cell embryos. Notably, there was an upregulation of totipotency markers such as *Zscan4*, *MERVL*, *Zfp352*, *Tcstv1*, and *Tcstv3*, while major pluripotency markers such as *Oct4*, *Sox2*, and *Nanog* were downregulated. Notably, the injection of single TPS cells into eight-cell embryos resulted in the formation of chimeric blastocysts comprising TPS cell-derived EPI and trophectoderm. These chimeric blastocysts further developed in vivo into chimeric embryos (E18.5) exhibiting a TPS cell-derived placenta. Based on the notion that the FGF, Bmp, and Yap signaling pathways are crucial for pre-implantation embryo development, particularly in the formation of the extraembryonic lineage [40,137,138,139], Xu et al. seeded TPS cells into AggreWell and treated them with bFGF, FGF4, Bmp4, and LPA for 4–6 days. The resulting blastoids displayed a great efficiency in cavity formation, exhibited transcriptomic similarity to E4.5 blastocysts in vitro, and demonstrated the capacity to induce implantation and decidualization in vivo, similar to E6.5 natural embryos, as evidenced by the PTGS2 in TPS-derived decidua [18].

In 2022, Yang et al. endeavored to remodel the chromatin to surmount the barriers of the pluripotency-to-totipotency transition. The key events that occur during the loss of totipotency are associated with nuclear remodeling, the clustering of pericentromeric heterochromatin leading to the formation of chromocenters, and the disappearance of broad H3K4me3 [19,124]. Yang et al. targeted DOT1L (a histone lysine methyltransferase) and KDM5B (a lysin demethylase). They found that the inhibition of DOT1L upregulates the expression of MERVL and other totipotent genes and induces 2C-like chromatin remodeling by disrupting the chromocenter, while the inhibition of KDM5B promotes the remodeling of two cell-specific broad H3K4me3 domains [140]. Consequently, they derived totipotent-like stem cells (termed TLSCs) from ESCs and early pre-implantation embryos (two-, four-, and eight-cell-stage embryos) using a TLSC medium containing interleukin 6 (IL6), soluble IL6 receptor (sIL-6R), SGC0946 (DOT1L inhibitor), and AS8351 (KDM5B inhibitor). IL6 and sIL-6R were included in the TLSC medium because these genes are highly expressed in both zygotes and two-cell embryos [19]. The established TLSCs expressed high levels of totipotency-specific genes and downregulated the expression of the pluripotency markers typically seen in TPS cells. When TLSCs were cultured in blastoid culture conditions [72,128,130,141], they formed aggregates recapitulating compaction and polarization that follow the ICM and TE specifications. The resulting TLSC-blastoids could be implanted into the uterus of pseudopregnant mice with more than 35% efficiency to form decidua, where a degraded embryo structure was observed, indicating the limited capacity of TLSCs to develop into a complete organism due to their restricted developmental potential [19].

Totipotent-like stem cells were also derived through the repression of spliceosome, which are ribonucleoprotein complexes that induce splicing and the maturation of messenger RNA [142,143]. It was observed that suppressing the expression of splicing factors in mouse ESCs led to the activation of totipotent marker genes [144]. Shen et al. attempted to convert pluripotent mouse ESCs to totipotent blastomere-like cells (TBLCs) through spliceosomal repression by pladienolide B (PlaB) [20], a naturally occurring antitumor analog that specifically binds to splicing factor SF3B [145]. The researchers found that reprogramming ESCs through spliceosomal repression resulted in the downregulation of pluripotency genes while activating totipotent genes. The derived TBLCs exhibited transcriptional characteristics similar to those of two- and four-cell blastomeres. Shen et al. confirmed the differentiation potential of TBSCs into both embryonic and extraembryonic lineages through chimera formation analysis. Later on, Zhang et al. demonstrated that TBLCs could efficiently form blastoids in a 3D differentiation culture system (AggreWell plates) with 80% efficiency [21]. Among the TBLC-blastoids, 71.3% expressed all three lineage markers (EPI, TE, and PrE). Although the efficiency was lower, blastoids could also be generated from a single TBLC. After being cultured in vitro for 6 days, around 10% of the TBLC-blastoids developed into egg cylinder-like structures. Additionally, when implanted into the uteruses of pseudopregnant mice, TBLC-blastoids formed deciduae, but approximately 95% of them remained empty without developing embryos. Furthermore, TBLC-blastoids displayed proportions of TE- and EPI-like cells that were comparable to those seen in natural blastocysts, but they exhibited fewer PrE-like cells. Therefore, achieving appropriate ratios of PrE and TE cells in TBLC-blastoids may be crucial for generating fully functional embryoids [21].

## 6. Human Blastoid Formation and In Vitro Implantation Development

**Figure 6:**
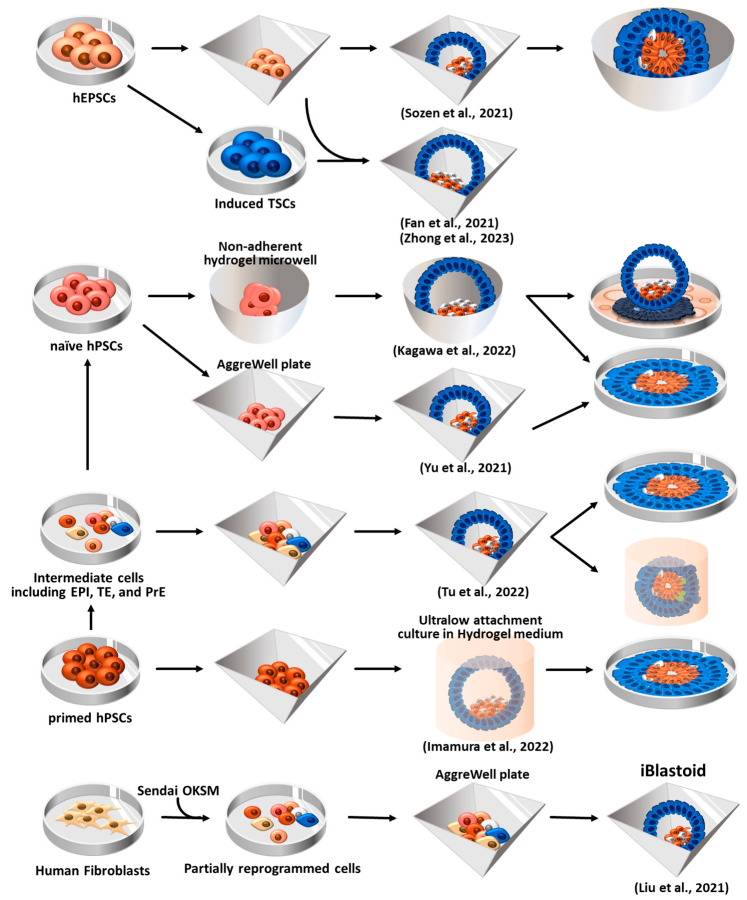
Human blastoid formation using hEPSCs and hPSCs and via somatic cell reprogramming. The resulting human blastoids are similar to natural blastocysts and exhibit the developmental potential to form structures resembling amniotic cavities and yolk sacs. hEPSCs: human expanded potential stem cells; hPSCs: human pluripotent stem cells; TSCs: trophoblast stem cells; EPI: epiblast; TE: trophectoderm; PrE: primitive endoderm; OKSM: Oct4, Klf4, Sox2, and c-Myc; iBlastoid: induced blastoid [24,25,26,27,28,150,151,152].

Since human ESCs (hESCs) and hiPSCs have the ability to differentiate into all cell types of the body, they have been used as in vitro models for studying early human development [146]. However, these models have limitations in fully replicating the blastocyst stage and providing comprehensive insights into human post-implantation development. Although notable advancements have been made in studying human embryonic development ex utero through in vitro fertilization and the culturing of donated blastocysts for research purposes [147,148,149], the availability of donated human embryos is restricted, and ethical challenges, as well as legal restrictions, impose significant constraints on their utilization in research. Hence, it is necessary to develop an in vitro model that can faithfully recapitulate pre- and post-implantation human embryonic development. In recent studies, human blastoids have been generated not only from human pluripotent stem cells (ESCs and iPSCs) but also from somatic cells through direct reprogramming, as shown in Figure 6, presenting a promising tool for understanding early human development [24,25,26,27,28].

### 6.1. Blastoid Formation Using Human EPSCs (hEPSCs)

Sozen et al. attempted to generate human blastoids using hEPSCs that were converted from human pluripotent stem cells (hPSCs) [25,128,131]. The converted hEPSCs formed dome-shaped colonies after more than five passages using a previously established LCDM medium [131]. After seeding the hEPSCs (four to five cells) in AggreWell 400, the hEPSCs readily formed aggregates. To facilitate aggregate formation, a medium containing Bmp4, CHIR99021 (Wnt agonist), FGF2, and Y-27632 (ROCK inhibitor) was employed. Additionally, A83-01 (ALK5 kinase inhibitor) was used to promote TE differentiation [153]. Within the initial 48 h of 3D culture, the enrichment of F-ACTIN and PARD6 (a polarity marker gene) at the apical surface of the structures was observed, confirming the occurrence of polarization. On day 6 of culture, blastocyst-like structures comprising three lineages–EPI, PrE, and TE—were formed. Moreover, upon subjecting these blastoids to extended culture in an IVC medium [147], they were able to form small lumens. However, the expression of certain TE markers became impaired as development progressed in vitro, and defects in aggregate formation were observed during the latter stage of the cavity formation process. To overcome defective TE formation, Sozen et al. combined hEPSCs with human TSCs (hTSCs) to generate blastoids, which was proposed in mouse blastoid formation [130]. Unlike in mice, the hTSCs were unable to compensate for TE lineage differentiation during hEPSC-derived blastoid formation.

Fan et al. generated human blastoids by combining hEPSCs with hEPSC-derived TE-like cells (a two-step induction protocol) [28]. The EPSCs, which were converted from hiPSCs, were treated with Bmp4 for 3 days to generate TE-like cells. These TE-like cells were then mixed with hEPSCs in AggreWell plates. By days 5–6, approximately 1.9% of the mixture had developed into blastoids exhibiting a diameter similar to E6 natural human blastocysts. The hEPSC-derived blastoids cultured through an IVC system [17,149] further developed beyond implantation and formed structures resembling 7–14 dpf (day post-fertilization) IVC natural embryos.

Zhong et al. used parthenogenetic hEPSCs (Pg-hEPSCs) that had been converted from parthenogenetic hESCs for the generation of human blastoids [26]. They also used the two-step induction protocol previously employed by Fan et al. [28]. Pg-hEPSC-derived TE-like cells were mixed with Pg-hEPSC in AggreWell plates, resulting in the formation of blastoids that resembled biparental hEPSC-derived blastoids comprising three-lineage cell types. Given that Pg-hESCs exclusively possess the maternal genome, Pg-hEPSCs blastoids can serve as an ideal model for investigating human diseases with parent-of-origin effects and exploring the function of imprinted genes during embryonic development [26].

### 6.2. Human Blastoid Induction via the Reprogramming of Fibroblasts

In addition to using previous approaches that involve using a combination of three blastocyst-derived stem cell types and using totipotent stem cells, Liu et al. developed a novel approach to generate induced blastoids (iBlastoids) from somatic cells through a reprogramming strategy [24]. EPI, TE, and PrE cells emerged by day 21 during the reprogramming of the fibroblasts under the fibroblast medium condition [154]. During the reprogramming process (by day 21), intermediate cells were transferred to AggreWell plates to induce aggregate formation, and the aggregates were then cultured for 7 days in a medium that supported early feature development. On day 6, these aggregates formed 3D blastocyst-like structures (iBlastoids). The generated iBlastoids were transcriptionally similar to natural blastocysts, and the area and diameter closely resembled those of E5–7 natural blastocysts. In vitro attachment assays [147,149] also confirmed that the iBlastoids could recapitulate the early developmental process. Over 90% of the iBlastoids attached within 24 h, exhibiting increased size, flattening, and the formation of outgrowths that resemble those observed in natural blastocysts. On day 3 of attachment, the iBlastoids displayed the polarization of Epi-like cells and the formation of a proamniotic-like cavity. This aligns with a previous report that indicated that EPI cells in human blastocysts undergo polarization and form a proamniotic-like cavity upon in vitro attachment [147]. In addition, trophoblast lineages, including syncytiotrophoblasts and extravillous cytotrophoblasts, were observed in the in vitro implantation iBlastoids, and this was accompanied by a 10-fold increase in the concentration of hCG (human chorionic gonadotropin) in the culture medium after 4.5 days of attachment. iBlastoids derived from somatic cells rather than stem cells offer the potential for broader utilization in studying the in vitro pre-implantation and peri-implantation stages of human embryogenesis.

### 6.3. Human Blastoid Formation from Primed and Naïve hPSCs

ESCs exist in at least two states that exhibit transcriptional similarities to that of the distinct stages of embryonic development. Following the fertilization of the egg by a sperm, the earliest human cells, which commit to forming the embryo, undergo a transformation into naïve pluripotent hESCs. Subsequently, when the embryo implants into the uterus, the naïve pluripotent hESCs within the embryo become ‘primed’ [155]. Naïve pluripotent hESCs exhibit a developmental resemblance to the embryonic epiblast at an earlier stage compared to ‘primed’ pluripotent hESCs [156]. While primed pluripotent hESCs exhibit restricted differentiation potential into the embryonic lineage, naïve pluripotent hESCs demonstrate a more versatile differentiation potential, encompassing both embryonic and extraembryonic (TE and PrE) lineages [155,157,158]. Thus, Yu et al. attempted to generate human blastoids using naïve hPSCs (both hESCs and hiPSCs) [27] that had been cultured in 5i/L/A medium [159]. The naïve hiPSCs were seeded into AggreWell plates and subjected to sequential induction, first towards the hypoblast lineage (via the treatment of FGF2, activin A, and CHIR9902) and subsequently towards the trophoblast lineage (via the activation of TGFβ-SMAD and WNT-β-catenin signaling in 50% 5i/L/A medium). This sequential method yielded human blastoids that closely resembled E6 natural blastocysts. Furthermore, after being cultured under IVC culture conditions for 4 days, the blastoids formed an amniotic cavity-like structure and a yolk sac-like cavity, which are features of peri-implantation human embryos.

Kagawa et al. also used naïve hPSCs to generate human blastoids [150]. Primed ESCs were converted to naïve ESCs using PXGL medium as described in a previously published study [160,161]; the PXGL medium contained PD0325901 (ERK inhibitor), XAV-939 (Wnt signaling inhibitor), Gö 6983 (PKC inhibitor), and hLIF (human leukemia inhibitory factor). The naïve PSCs were aggregated in non-adherent hydrogel microwells and cultured in PALLY medium supplemented with PD0325901, A83-01 (TGFβ family receptors inhibitor), LPA (Hippo pathway inhibitor), hLIF (STAT activator), and Y-27632, resulting in blastoids with an efficiency of over 70% at 4 days of culture. The cell number and size of the blastoids were within the ranges observed in E5–7 blastocysts. Yanagida et al. also reported human blastoids derived from naïve hESCs cultured in PXGL medium. These human blastoids could simulate the early event of implantation in vitro when cultured on the endometrial cell layer [150] or Geltrex-coated dishes [24,25,27,28,162]. In vitro, the implanted human blastoids formed syncytiotrophoblast, yolk sac, and amniotic cavities [24,25,27,28,162].

A recent study reported that EPI, TE, and PrE cells appeared during the conversion of primed hPSCs into naïve hPSCs [163]. Tu et al. attempted to convert primed hPSCs into a naïve state using 5i/L/A culture conditions [159] and to capture three lineage cell types to generate blastoids [151]. They found that a high proportion of EPI-like (35.5%), TE-like (17.0%), and PrE-like (28.8%) subpopulations were present in intermediate cells at day 8 of naïve conversion. Thus, Tu et al. seeded the intermediate cells (at day 8) into an AggreWell plate and induced blastoid formation. The resulting human blastoids exhibited transcriptional similarities to E6 natural blastocysts. Moreover, when the blastoids were subjected to in vitro attachment (to achieve post-implantation development) [147,149], they formed flattened structures that underwent expansion and further developed to form the trophoblast-like lineage, including syncytiotrophoblast-, extravillous cytotrophoblast-, proamniotic cavity-, and yolk sac-like structures.

Imamura et al. noted that human blastocysts are surrounded by a glycoprotein layer—zona pellucida (ZP)—and that primed hPSCs can be converted to a naïve pluripotent state through 3D culture in AggreWell plates [164]. In their study, aggregates of primed hPSCs were transferred to ultralow attachment culture dishes in hydrogel (HG) medium, which provides an environment for the ZP layer and converts to a naïve hPSC state with the aim of inducing blastoid formation [152]. Approximately 14.1% of the structures formed exhibited features similar to E5–7 blastocysts. These HG-blastoids were able to mimic the cell distribution of the three lineages found in natural blastocysts. Moreover, when cultured in vitro, the HG-blastoids formed a proamniotic-like cavity and cytotrophoblast-like cells that showed partial functionality. However, the number of TE- and PrE-like cells in these blastoids was relatively low compared to that of natural blastocysts. Additionally, the blastoid formation efficiency of this study remained low compared to other methods using naïve hPSCs [152].

In summary, human blastoids can be generated through various methods, including the 3D culturing of naïve hPSCs, conversion of primed and naïve hPSCs, and somatic cell reprogramming [24,25,26,27,28]. Human blastoids exhibit many features of natural blastocysts, including morphological and architectural similarities, the ability to undergo in vitro peri-implantation development, and the capability to derive three stem cell types (ESCs, XENCs, and TSCs) [27,150,151,152]. This suggests that human blastoids are useful in in vitro models designed for studying pre- and peri-implantation development in humans. However, it is important to note that, compared to natural blastocysts, many of these blastoids may display structural abnormalities due to incorrect compositions of TE and EPI and that, over time, the efficiency of in vitro 3D culture tends to decline [151].

Although human blastoids have shown promise in the study of human embryo development, infertility, teratogenicity, and disease without using natural embryos, the use of human blastoids for research and clinical application may raise important ethical issues. Some people argue that the 14-day rule, which restricts research on human embryos up until a maximum of 14 days or until the appearance of the primitive streak [29,165], is also applicable for regulating human synthetic embryos. However, in 2021, the International Society for Stem Cell Research (ISSCR) decided to remove the ban on the “culture of human embryos beyond 14 days or primitive streak formation”. Instead, the ISSCR has classified human embryo models into two groups: “non-integrated” models that partially mimic early embryonic development but lack the potential for substantial development due to the lack of extraembryonic tissues and “integrated” models that include all embryonic structures, along with extraembryonic tissues, necessitating more rigorous monitoring [166]. There are conflicting views on the regulation of the human blastoids, as they do not fully replicate the developmental stage of blastocysts and are not functionally equivalent to human embryos. Some argue that blastoids may not require the same level of regulation [167,168], while others believe that as they advance in morphology and functionality, their closer resemblance to human embryos will necessitate similar regulatory measures [169]. The first viewpoint avoids using natural blastocysts but raises ethical concerns if blastoids become functionally equivalent to blastocysts in the future. The second viewpoint is safer from a moral perspective but limits research. Despite the fact that, currently, there is insufficient evidence to determine blastoids’ functional equivalence to natural blastocysts, it may be premature to exclude them from ethical scrutiny and regulation. Another viable option to address ethical concerns is to focus on gastruloid research, as these 3D models can partially mimic late-stage development beyond the 14-day restriction without forming a complete organism.

## 7. Gastruloids

Blastoid and embryoid models aim to recapitulate in vivo embryogenesis through the co-culture of stem cells originating from early-stage embryos. Other approaches have been pursued to simulate early embryos and gastrula in vitro. In 2014, Martinez Arias et al. generated a 3D structure called a “gastruloid” by aggregating a small number of ESCs and inhibiting several signaling pathways [4]. Gastruloids mimic the embryonic gastrulation process, including axis organization and germ layer specification, without the presence of extraembryonic lineages. Aggregates of approximately 300 mouse ESCs were treated with Activin A, CHIR99021 (agonists of the Wnt/β-catenin signaling), and BMP (a mesoderm inducer) to initiate primitive streak (PS) formation. Following a treatment period of 48–72 h and an analysis period that spanned up to 5 days, the resulting gastruloids exhibited significant features that resembled early mouse embryos, including symmetry breaking, axial organization, the specification of germ layers, the initiation of gastrulation, and the elongation of the axis [4]. In a follow-up study, AP polarization [170], the dorsal–ventral (D-V) and medio–lateral (M-L) axes, and left-right (L-R) asymmetry was confirmed in gastruloids through the expression analysis of respective markers [171]. Although gastruloids demonstrated a superior capacity to specify most embryonic axes compared to single-lineage organoids, they did not fully replicate the morphological features or transcriptional signatures observed during late embryonic development.

Van den Brink et al. developed the gastruloid formation protocol to induce organogenesis and the formation of anterior neural outgrowths [172]. By employing single-cell RNA sequencing and spatial transcriptomics techniques, they found that gastruloids exhibited gene expression patterns characteristic of axis formation and somitogenesis, closely resembling those observed in mouse embryos [172]. Furthermore, when embedded in a Matrigel, these gastruloids formed somites along the A-P axis [172]. A “trunk-like structure” consisting of the neural tube, gut, and somites was also generated through the embedding of four-day-old gastruloids within a Matrigel matrix [173]. The treatment of early-stage EPI-like aggregates with the Wnt signaling inhibitor XAV could promote the formation of anterior neural tissue in gastruloids [173]. Rossi et al. generated beating gastruloids through the treatment of cardiogenic factors (bFGF, ascorbic acid, and VEGF) [174]. Notably, in vitro cardiogenesis faithfully reproduces the critical features of heart development, including the formation of a crescent-like structure, the vascular networks, and the interactions with surrounding tissues [174]. Gastruloid formation mainly relies on the self-organization properties of PSC and their response to signaling pathways that induce the differentiation in different germ layers. To enhance the structural complexity and functional diversity of gastruloids, Xu et al. introduced a experimentally controlled method that does not solely rely on the self-organization of aggregates [175]. They integrated ESC aggregates with BMP4-treated aggregates that express WNT3 and NODAL at a later stage. The activation of WNT3 and NODAL signaling in the aggregates promoted the formation of mesoderm, followed by a notochord-like structure, neural plate, neural tube, axial mesoderm, and endodermal and mesodermal derivatives. Based on the knowledge that reducing cell SUMOylation promotes reprogramming to a pluripotent state and trans- and terminal differentiation [176,177,178], Cossec et al. conducted an experiment to convert mouse ESCs to a 2C-like state using ML-792, a SUMOylation inhibitor. As a result, they obtained three cell types capable of self-assembly into adherent spheroids, leading to the formation of self-organized embryo-like structures (ELSs) with head- and trunk-like structures, achieving an efficiency of 74%. The ELSs also underwent a gastrulation process similar to those observed during the post-implantation stage of embryo development. However, ELSs displayed defective development in PGCs and placental lineages, highlighting the need to address these issues and improve the efficiency and refinement of gastruloid formation [22].

In studies exploring human gastruloids, it has been observed that the treatment of human ESCs with the Wnt agonist Chiron, both before and after aggregation, leads to the formation of gastruloids exhibiting spatial polarization and the derivates of three germ layers [179]. Notably, these gastruloids lack the development of the anterior regions where the brain forms and lack extra-embryonic tissue, thereby circumventing the ethical concerns associated with human embryo research [179]. Olmsted et al. employed a sequential culture method to generate gastruloids by first 2D culturing hiPSC treated with CHIR99021 and FGF2 and then transferred them into suspension culture with a medium containing FGF2, IGF-1, HGF, and Y-27632 [180]. The resulting human gastruloids exhibited a remarkable resemblance to the early stages of neurodevelopment and displayed extensive connections with other organ systems such as cardiac lineages, forming neuro-cardiac gastruloids [180]. This study provides a deeper understanding of multi-lineage development during early embryonic organogenesis [181].

## 8. Conclusions

In summary, combining the three blastocyst-derived stem cell types (ESCs, XENCs, and TSCs) or using totipotent-like stem cells alone, along with the diverse manipulation of developmental signaling molecules, results in the formation of synthetic embryos, including blastoids and embryoids. The blastoid model demonstrates the ability to mimic key aspects of pre-implantation development, such as polarization and cavitation. Through extended culture in vitro, synthetic embryos recapitulate post-implantation development both in embryonic and extraembryonic tissues. However, limitations in accurately mimicking implantation and the development of extraembryonic lineages need to be overcome to create a more comprehensive organism model. By refining culture systems, incorporating modeling mechanical environments, and developing living cell-based maternal implantation models, we can overcome these limitations, unlock deeper insights into the complexities of early development, and open a new field for research with a living, comprehensive organism model.

These synthetic embryo models offer a research tool for studying early developmental events, providing insights into lineage specification, tissue patterning, and organogenesis. Furthermore, they hold potential for clinical applications, including disease modeling, drug screening, and regenerative medicine. However, ethical considerations must be balanced with the promise these models hold for advancing scientific knowledge and improving human health. Gastruloids can serve as an alternative model for recapitulating the embryonic gastrulation process without forming extraembryonic lineage tissues, thereby offering a viable option to address the aforementioned ethical concerns when applied in humans.

## Data Availability

Not applicable.
